# Phylogeny, diversification, and biogeography of a hemiclonal hybrid system of native Australian freshwater fishes (Gobiiformes: Gobioidei: Eleotridae: *Hypseleotris*)

**DOI:** 10.1186/s12862-022-01981-3

**Published:** 2022-03-02

**Authors:** Christine E. Thacker, James J. Shelley, W. Tyler McCraney, Mark Adams, Michael P. Hammer, Peter J. Unmack

**Affiliations:** 1 Vertebrate Zoology, Santa Barbara Museum of Natural History, 2559 Puesta del Sol, Santa Barbara, CA 93105 USA; 2 Research and Collections, Department of Ichthyology, Natural History Museum of Los Angeles County, 900 Exposition Blvd., Los Angeles, CA 90007 USA; 3 School of BioSciences, University of Melbourne, Melbourne, VIC 3010 Australia; 4 Department of Environment, Land, Water and Planning, Arthur Rylah Institute for Environmental Research, 123 Brown Street, Heidelberg, VIC 3084 Australia; 5 Department of Ecology and Evolutionary Biology, University of California, Los Angeles, 612 Charles E. Young Drive South, Box 957246, Los Angeles, CA 90095-7246 USA; 6 Evolutionary Biology Unit, South Australian Museum, North Terrace, Adelaide, SA 5000 Australia; 7 School of Biological Sciences, University of Adelaide, Adelaide, SA 5005 Australia; 8 Museum and Art Gallery of the Northern Territory, GPO Box 4646, Darwin, NT 0801 Australia; 9 Centre for Applied Water Science, Institute for Applied Ecology, University of Canberra, Canberra, ACT 2617 Australia

**Keywords:** Eleotridae, Phylogeny, Evolution, Hybrid, Hemiclone, Genomics, Climate, Biogeography

## Abstract

**Background:**

Carp gudgeons (genus *Hypseleotris*) are a prominent part of the Australian freshwater fish fauna, with species distributed around the western, northern, and eastern reaches of the continent. We infer a calibrated phylogeny of the genus based on nuclear ultraconserved element (UCE) sequences and using Bayesian estimation of divergence times, and use this phylogeny to investigate geographic patterns of diversification with GeoSSE. The southeastern species have hybridized to form hemiclonal lineages, and we also resolve relationships of hemiclones and compare their phylogenetic placement in the UCE phylogeny with a hypothesis based on complete mitochondrial genomes. We then use phased SNPs extracted from the UCE sequences for population structure analysis among the southeastern species and hemiclones.

**Results:**

*Hypseleotris cyprinoides*, a widespread euryhaline species known from throughout the Indo-Pacific, is resolved outside the remainder of the species. Two Australian radiations comprise the bulk of *Hypseleotris*, one primarily in the northwestern coastal rivers and a second inhabiting the southeastern region including the Murray–Darling, Bulloo-Bancannia and Lake Eyre basins, plus coastal rivers east of the Great Dividing Range. Our phylogenetic results reveal cytonuclear discordance between the UCE and mitochondrial hypotheses, place hemiclone hybrids among their parental taxa, and indicate that the genus *Kimberleyeleotris* is nested within the northwestern *Hypseleotris* radiation along with three undescribed species. We infer a crown age for *Hypseleotris* of 17.3 Ma, date the radiation of Australian species at roughly 10.1 Ma, and recover the crown ages of the northwestern (excluding *H. compressa*) and southeastern radiations at 5.9 and 7.2 Ma, respectively. Range-dependent diversification analyses using GeoSSE indicate that speciation and extinction rates have been steady between the northwestern and southeastern Australian radiations and between smaller radiations of species in the Kimberley region and the Arnhem Plateau. Analysis of phased SNPs confirms inheritance patterns and reveals high levels of heterozygosity among the hemiclones.

**Conclusions:**

The northwestern species have restricted ranges and likely speciated in allopatry, while the southeastern species are known from much larger areas, consistent with peripatric speciation or allopatric speciation followed by secondary contact. Species in the northwestern Kimberley region differ in shape from those in the southeast, with the Kimberley species notably more elongate and slender than the stocky southeastern species, likely due to the different topographies and flow regimes of the rivers they inhabit.

## Background

Australia is home to a unique and unusual fauna of freshwater fishes. Except for two species of Osteoglossidae, almost all Australian freshwater fishes are derived from marine ancestors, a consequence of the continent’s long isolation from other Gondwanan landmasses [1]. This isolation has yielded a fauna composed nearly entirely of Acanthopterygiian fishes, rather than the primary freshwater Ostariophysi so common elsewhere [2]. The Australian continent is generally arid, particularly inland across the center and the west, and includes relatively few aquatic habitats compared to other continents [3]. Two major and one minor inland river systems drain the eastern half of the continent. The Murray–Darling Basin in the southeast drains into the Southern Ocean near Adelaide. Immediately west of the Murray–Darling Basin is the small endorheic Bulloo-Bancannia Basin, which starts in western Queensland and terminates in northwestern New South Wales. In central Australia, the endorheic Lake Eyre Basin includes four larger river systems: Cooper Creek, and Diamantina, Georgina, and Finke rivers, which have highly intermittent flows. Additionally, the continent is ringed by shorter coastal rivers in the west, north, and east that drain mountain ranges or tablelands and flow to the sea. Arrayed throughout these systems are just over 300 described species of freshwater fishes, a tiny number compared to freshwater ecosystems of similar size on other continents [1], but likely a significant underestimate of the true number due to a lack of taxonomic focus [4–6]. The fauna is notable for the absence of Cypriniformes and Characiformes, and for the presence of endemic radiations of several clades of Atheriniformes and Gobiiformes, in particular, the gudgeons.

Gudgeons are Gobioidei in the families Butidae and Eleotridae, and both families are represented in Australian freshwaters by several genera. Among Eleotridae, the colourful spotted *Mogurnda* species have radiated throughout the north and east of Australia, as well as in nearby New Guinea [7]. *Philypnodon* and *Gobiomorphus* are each represented by two described species in eastern Australia. *Philypnodon* is endemic to Australia, and *Gobiomorphus* includes an additional seven species known from New Zealand [1, 8]. Recent evidence suggests that Australian *Gobiomorphus* are more closely related to *Philypnodon* than their New Zealand congeners [9]. The most speciose eleotrid radiation in Australia is the genus *Hypseleotris*, containing eight described and several undescribed species, all but two of which are restricted to Australian fresh waters (one *Hypseleotris* species, *H. cyprinoides*, is distributed widely throughout the Indo-Pacific and is not found in Australia). The Australian gudgeon genera are placed throughout the phylogeny of Eleotridae and Butidae, indicating that Australian freshwaters have experienced multiple independent gudgeon invasions since the mid-Oligocene [10, 11].

*Hypseleotris* species are also known as carp gudgeons due to their laterally compressed body shapes and pointed snouts that give them an overall resemblance to some cyprinids. Unusually for gudgeons, *Hypseleotris* species are not strictly benthic and are instead often found hovering above the substrate near aquatic plants or other cover. Sexual dimorphism is often pronounced during the breeding season, when males have intensified body and fin colors and develop slight to prominent humps on the forehead while females have enlarged and often vibrantly colored bellies [1, 12]. *Hypseleotris* species are common in Australian waterways but the taxonomy of the various species has been difficult to disentangle, due to the overall similarity of many species as well as the presence of stable hemiclonal hybrid lineages among several taxa in southeastern Australia [13, 14].

**Fig. 1:**
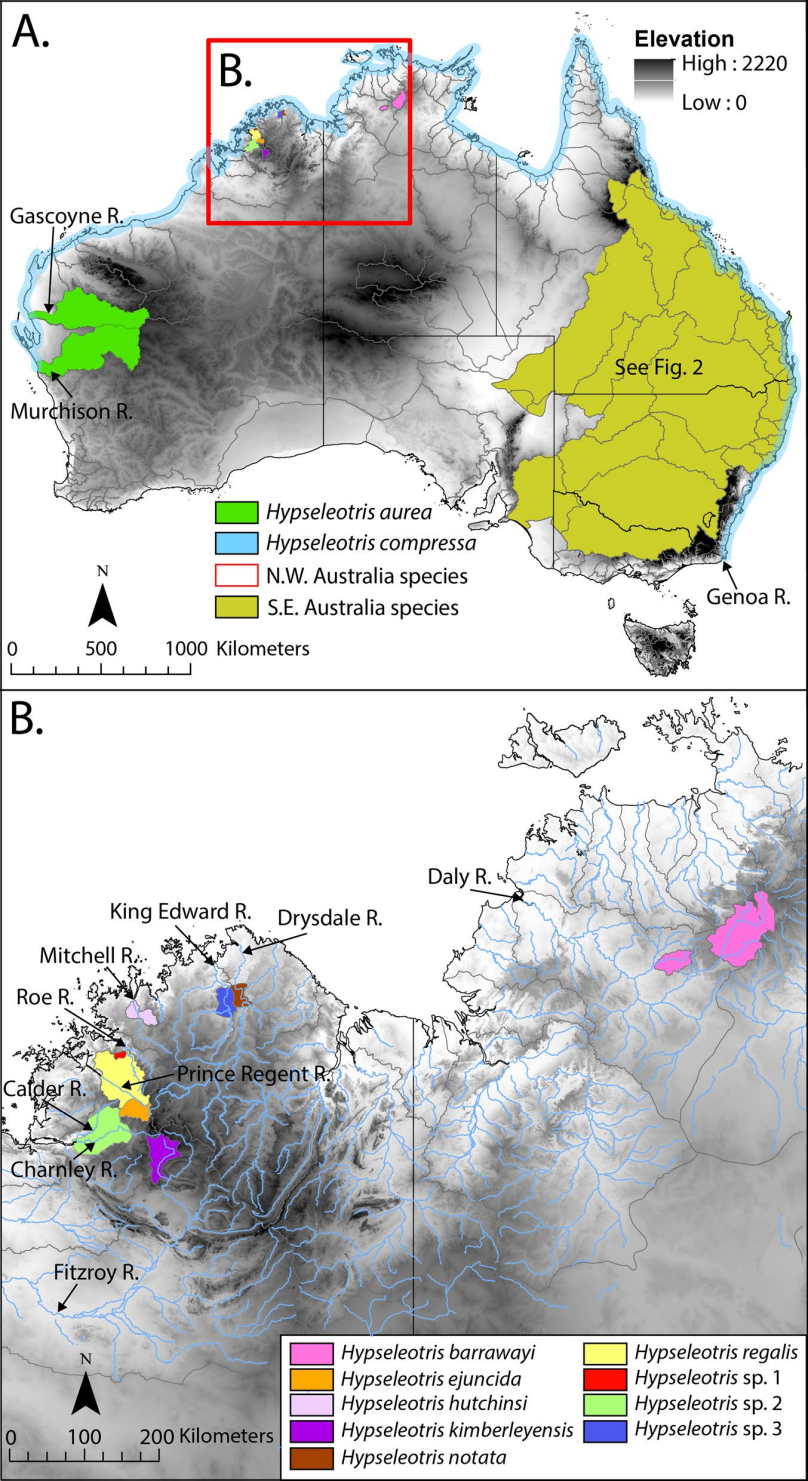
Distributions of **A** *Hypseleotris aurea* and *H. compressa*, and **B** *Hypseleotris* species from northwestern Australia, with major river drainages indicated

*Hypseleotris* in Australia have regionally restricted distributions, with the exception of one widespread euryhaline species, *H. compressa*, which inhabits coastal rivers in the Pilbara region (WA), from the Murchison River across northern Australia and down the east coast to the Genoa River on the Victorian border. This species shows the strongest sexual dimorphism (males can turn bright orange-red overall) and larger adults are deep bodied. *Hypseleotris compressa* is also known from southern New Guinea and islands in the Torres Strait; a distribution map for *H. compressa* in Australia is given in Fig. 1.

Along the west and north coasts of Australia, seven endemic *Hypseleotris* species are known. *Hypseleotris aurea* occurs in the Gascoyne and Murchison rivers of the Pilbara region; the distribution for this species is given in Fig. 1. An additional six species (three described and three undescribed) are known from very restricted distributions in the Kimberley Plateau region including the Prince Regent, Barnett, Calder, Charnley, Carson and Roe rivers [12, 15]. In that same region (Mitchell and Drysdale rivers) two species of the closely aligned genus *Kimberleyeleotris* also occur*. Kimberleyeleotris* is morphologically similar to *Hypseleotris* found in the Kimberley region, and was described as differing from them in having fewer scales, teeth on the vomer, and slight differences in head papillae pattern and mouth shape [15, 16]. *Hypseleotris barrawayi* occurs in the adjacent Arnhem Plateau, Northern Territory, restricted to the upper Daly River system [17]. *Kimberleyeleotris* and the six Kimberley *Hypseleotris* are collectively slender bodied, with *H. aurea* and *H. barrawayi* slightly deeper (intermediate) bodied. A distribution map for the northwestern species is given in Fig. 1.

**Fig. 2:**
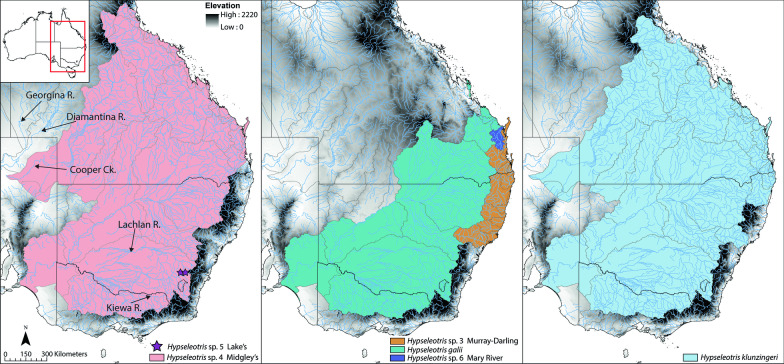
Distribution of southeastern *Hypseleotris* species, with major river drainages indicated

Two described and four undescribed species inhabit southeastern Australian rivers: *H. galii*, *H. klunzingeri*, and the undescribed but well-known species *H*. sp. 3 Murray–Darling, *H*. sp. 4 Midgley’s, *H.* sp. 5 Lake’s, and *H.* sp. 6 Mary River [18–20]. There are two additional lineages within *H. klunzingeri* that likely warrant species status, but they are cryptic and have not been accorded common names (Unmack, unpublished data). Hybrid hemiclonal lineages have resulted from crosses among most of the southeastern species except *H. klunzingeri* [13, 14]. The southeastern species generally have stocky bodies, most pronounced with *H.* sp. 4 Midgley’s and *H.* sp. 5 Lake’s and their various hemiclones, and less so (intermediate) for the *H. klunzingeri* complex and *H. galii, H.* sp. 3 Murray–Darling, and *H*. sp. 6 Mary River [1, 20]. A distribution map for these species is given in Fig. 2, and all species names and their distributions are listed in Table 1.

**Table 1:** Species of *Hypseleotris* and their geographic ranges

Species	Range
*H.* sp. 3 Murray–Darling	Murray–Darling Basin drainages and East Coast drainages north of Mary River
*H. aurea*	Gascoyne and Murchison rivers, Western Australia
*H. barrawayi*	Upper Katherine and Edith rivers, Daly River system, Northern Territory
*H.* sp. 4 Midgley’s	Murray–Darling, Bulloo-Bancannia, and Lake Eyre basins, East Coast drainages between Tully and Brisbane rivers, upper Einasleigh River in Gulf of Carpentaria
*H. compressa*	Coastal drainages in western, northern, and eastern Australia, southern New Guinea
*H. cyprinoides*	Madagascar, South Africa, Indonesia, Oceania
*H. ejuncida*	Upper Prince Regent River, Kimberley region, Western Australia
*H. galii*	East Coast drainages south of the Mary River to the vicinity of the Hunter River
*H.* sp. 5 Lake’s	Two small tributaries to the Lachlan River, Murray–Darling Basin
*H. hutchinsi*	Mitchell River, Kimberley region, Western Australia
*H. kimberleyensis*	Barnett River Gorge in the upper Fitzroy River, Kimberley region, Western Australia
*H. klunzingeri*	Murray–Darling, Bulloo-Bancannia, Lake Eyre basins, East Coast drainages, Burdekin River, then between Herbert Creek and Clarence River, with a disjunct population further south in Hunter River
*H.* sp. 6 Mary River	Mary River, Queensland
*H. notata*	Lower Drysdale River, Kimberley region, Western Australia
*H. regalis*	Roe River and lower Prince Regent River, Kimberley region, Western Australia
*H*. sp. 1 Garimbu gudgeon	Above a waterfall on Garimbu Creek, Roe River, Kimberley region, Western Australia
*H*. sp. 2 Bachsten gudgeon	Charnley and Calder rivers, Kimberley region, Western Australia
*H*. sp. 3 King Edward gudgeon	Carson River, King Edward River, Kimberley region, Western Australia

*Hypseleotris* *hutchinsi* and *H. notata* were originally classified in the genus *Kimberleyeleotris*

A phylogeny of the genus [21] based on parsimony analysis of morphological characters and partial sequence of the mitochondrial ND2 gene demonstrated that the one widespread euryhaline *Hypseleotris* species that does not occur in Australia, *H. cyprinoides*, was synonymous with populations known by other species names: *H. tohizonae* in Madagascar, *H. dayi* in South Africa, *H. leuciscus* in Indonesia, and *H. guentheri* from islands of Oceania. That study also recovered a clade consisting of the species *H. compressa*, *H. aurea*, and the northwestern Australian species *H. barrawayi* (then undescribed), *H. ejuncida*, *H. kimberleyensis*, and *H. regalis*. A second, southeastern Australian clade comprised the species *H. galii*, *H.* sp. 3 Murray–Darling, *H.* sp. 4 Midgley’s, and *H.* sp. 5 Lake’s (all individuals denoted sp. 5 Lake’s in that analysis were hemiclone combinations with *H.* sp. 5 Lake’s as one parent). The other widespread southeastern species, *H. klunzingeri*, was placed outside those two clades with weak phylogenetic support. A later phylogeographic study, also using mitochondrial ND2 data but with greatly expanded intraspecific sampling and focusing on southeastern *Hypseleotris*, confirmed the grouping of *H. galii*, *H.* sp. 3 Murray–Darling, *H.* sp. 4 Midgley’s, and *H.* sp. 5 Lake’s, to the exclusion of *H. klunzingeri* and with some genetic mixing among the pairs *H. galii*/*H.* sp. 3 Murray–Darling and *H.* sp. 4 Midgley’s/*H.* sp. 5 Lake’s [22]. In that mitochondrial phylogeny, mixing among the taxa was due to the presence of hybrid hemiclonal lineages among southeastern Australian species [14, 23]. Phylogenetic studies based on mitochondrial data reveal only maternal inheritance patterns, and in particular, will not distinguish between species and interspecific hybrids.

In this study, we reexamine the phylogeny of *Hypseleotris* species using a source of abundant nuclear DNA sequence data: ultraconserved elements (UCEs) [24, 25]. UCE regions are distributed throughout the genome, and hundreds of loci may be amplified along with their more variable flanking sequences to provide phylogenetic resolution at both shallow and deep evolutionary timescales [26–28]. We include representatives of all *Hypseleotris* species, and for the species in southeastern Australia we sample not only the sexual species but also their hemiclonal hybrid lineages. We also include three individuals of undescribed *Hypseleotris* species from the Kimberley region along with another Kimberley species in a related genus, *Kimberleyeleotris hutchinsi*. Our goal is to reconstruct a robust hypothesis of phylogeny using nuclear genes representing a much larger and more complete dataset than has been used previously. We also infer phylogeny based on complete mitochondrial genomes assembled from off-target UCE reads to distinguish the maternal parents of the hemiclone hybrids. To identify incomplete lineage sorting among taxa, we construct a species tree from individual UCE gene trees using ASTRAL. We then construct a UCE phylogeny of only the pure *Hypseleotris* species (without hemiclone hybrids), calibrate this hypothesis using fossil-based legacy calibrations, and compare climactic history and geological variability across Australia to the phylogenetic patterns. The radiations of *Hypseleotris* in northwestern and southeastern Australia have arisen and diversified in environments that are physically disjunct as well as topographically distinct, and we use GeoSSE to perform range-dependent diversification analysis comparing speciation dynamics between the two radiations. We then use GeoSSE again to analyze just the northwestern species and *H. compressa* to assess support for diversification differences between clades in the western Kimberley region as compared to the eastern Kimberley and the Arnhem Plateau. Finally, we extract and phase SNP variants from the UCE sequences for the southeastern species and hemiclones, to evaluate the genetic composition of the hemiclones and compare levels of heterozygosity between the hemiclones and parental taxa.

## Results

### *Hypseleotris* phylogeny and mitonuclear discordance among the southeastern hemiclones

**Fig. 3:**
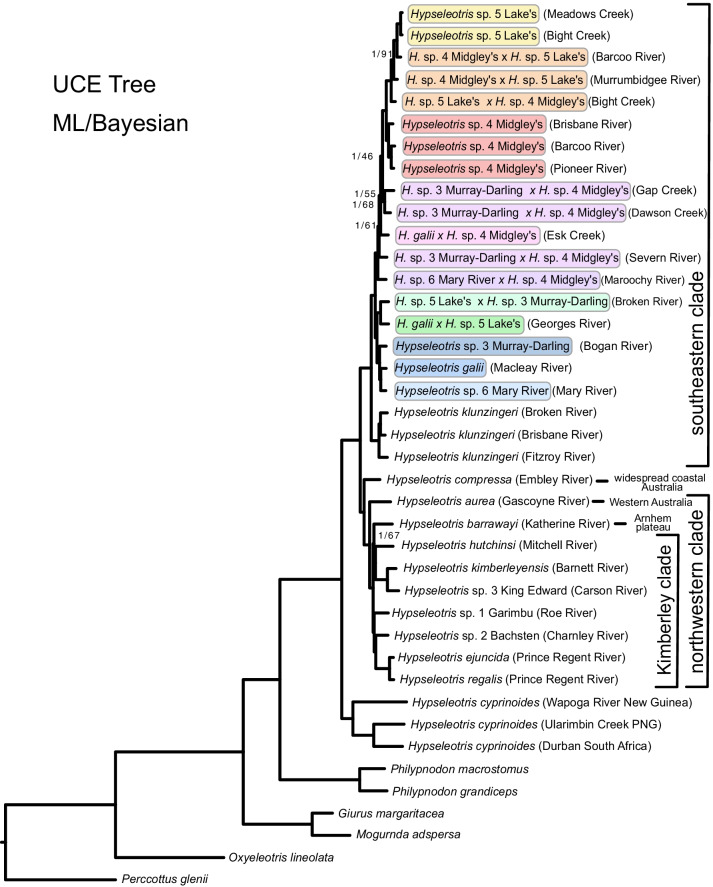
UCE hypothesis, based on both ML and Bayesian analyses. All nodes are supported at 1.0 posterior probability in the Bayesian hypothesis, and 95–100% bootstrap support in the ML hypothesis, except where indicated; at those nodes the posterior probability/bootstrap support are shown. Hemiclone-forming southeastern *Hypseleotris* species are bounded with colored boxes: yellow for *H.* sp. 5 Lake’s, orange for hemiclone pairings between *H.* sp. 4 Midgley’s and *H.* sp. 5 Lake’s, red for *H.* sp. 4 Midgley’s, shades of purple for the hemiclones of *H.* sp. 4 Midgley’s and species of the *H. galii* clade, shades of green for hemiclones of *H.* sp. 5 Lake’s and blue for species of the *H. galii* clade. The hemiclones are labeled with the maternal parent of the cross listed first. *Hypseleotris* individuals are labeled in parentheses with the locality where they were collected, not the entire range of the taxon

The phylogenetic hypothesis of *Hypseleotris* and outgroups based on analysis of UCE loci with both ML and Bayesian inference is given in Fig. 3. Our phylogeny agrees in many respects with that of [21], although with far better nodal support. The only weakly supported portions of our hypothesis are the nodes subtending some of the hemiclone hybrid taxa in the southeastern clade and the node separating *H. barrawayi* and *Kimberleyeleotris hutchinsi* in the northwestern clade. Nodes other than those are well-supported (95–100% bootstrap in the ML analysis and 100% posterior probability in the Bayesian analysis), even though many of the internodes within radiations are shallow.

Inside the outgroup Odontobutidae (*Perccottus glenni*) and Butidae (*Oxyeleotris lineolata*), we resolve the clade containing *Giurus margaritacea* and *Mogurnda adspersa* as the earliest diverging lineage within Eleotridae, congruent with [11]. We resolve the two *Philypnodon* species as sister to *Hypseleotris*, but caution that, due to the limited sampling of eleotrid genera, this placement does not indicate that *Philypnodon* is the closest relative to *Hypseleotris*. We confirm the monophyly of *Hypseleotris*, and that *H. cyprinoides* is the earliest diverging species, here represented by individuals from New Guinea and South Africa. These individuals are relatively divergent from one another, possibly indicating distinct lineages in geographically distant populations of *H. cyprinoides*. However, as discussed in [21], due to the depauperate sampling and lack of distinguishing morphological characters among the populations, we retain the single name *H. cyprinoides* for the clade.

Within the Australian *Hypseleotris* radiation, we recover two clades. The first is composed of the eastern and southern species, including *H.* sp. 4 Midgley’s, *H.* sp. 5 Lake’s, *H.* sp. 3 Murray–Darling, *H.* sp. 6 Mary River, *H. galii,* and *H. klunzingeri*. Here, in contrast to [21], we recover *H. klunzingeri* with its geographically close congeners, rather than outside the bulk of *Hypseleotris* species. We confirm the sister taxon pairings of *H.* sp. 4 Midgley’s/*H.* sp. 5 Lake’s and *H.* sp. 3 Murray–Darling/*H.* sp. 6 Mary River/*H. galii*, clades whose species hybridize with one another to form hybrid hemiclonal lineages [14]. The hemiclone hybrid individuals involving *H*. sp. 3 Murray–Darling*, H.* sp. 6 Mary River, or *H. galii* parents are resolved in a grade between the *H.* sp. 3 Murray–Darling/*H.* sp. 6 Mary River/*H. galii* clade and the crown group of *H*. sp. 4 Midgley’s, *H.* sp. 5 Lake’s, and their hybridogens.

The second Australian *Hypseleotris* clade mostly consists of a radiation in the north and west of Australia, and also includes the widespread *H. compressa* (present in coastal Australia and southern New Guinea). We recover *H. compressa* as the earliest diverging member of this clade, also in contrast to the earlier (weakly supported) resolution of [21]. Next diverging is *H. aurea*, from the Pilbara region of Western Australia, followed by eight species distributed in or near the Kimberley region. These include the named species *H. kimberleyensis, H. barrawayi*, *H. ejuncida*, and *H. regalis*, along with three undescribed species [12] and *Kimberleyeleotris hutchinsi*, a very similar gudgeon species. These relationships are in agreement, where they overlap, with those of [21]. The Kimberley species comprise two clades: one including *H. barrawayi*, *K. hutchinsi, H. kimberleyensis* and *H.* sp. 3 (King Edward gudgeon), and a second including *Hypseleotris* sp. 1 (Garimbu gudgeon), *H*. sp. 2 (Bachsten gudgeon), *H. ejuncida*, and *H. regalis* [12].

**Fig. 4:**
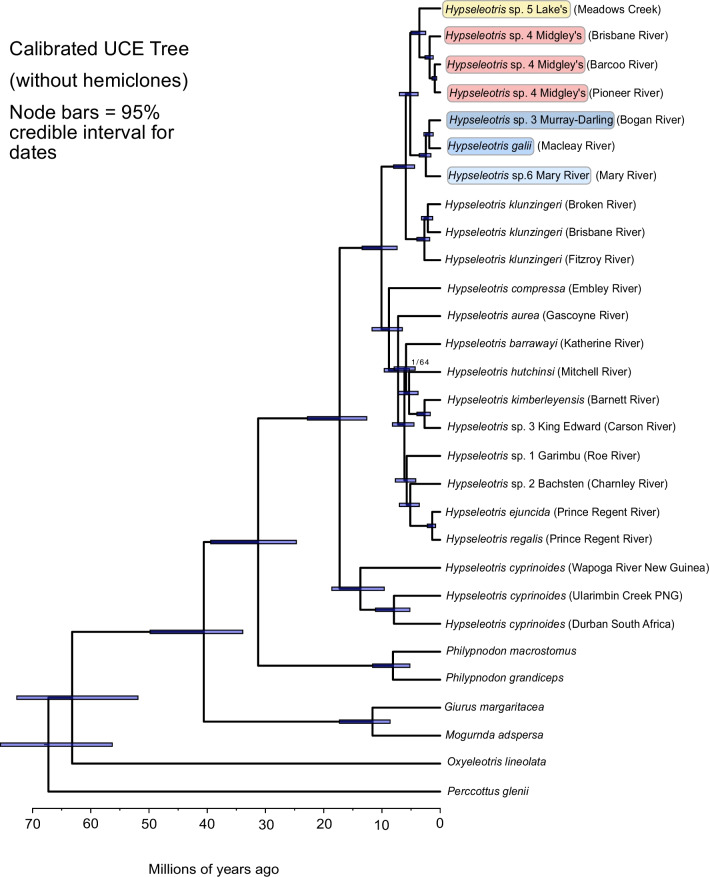
UCE hypothesis, dated with MCMC tree. All nodes are supported at 1.0 posterior probability in the Bayesian hypothesis, and 97–100% bootstrap support in the ML hypothesis, except where indicated; at those nodes the posterior probability/bootstrap support are shown. Bars on nodes are 95% highest posterior density intervals of age estimates shown in the lower scale. *Hypseleotris* individuals are labeled in parentheses with the locality where they were collected, not the entire range of the taxon. Colors and names for hemiclone-forming southeastern species are as denoted in Fig. 1

Inferred divergence times for *Hypseleotris* based on a separate phylogenetic analysis of UCE data without the hemiclone hybrids were consistent across the three MCMCTree runs and are shown in Fig. 4 (calibration analysis of the UCE matrix with the hemiclones included yielded similar results, although the inferred dates were slightly older). The crown age of *Hypseleotris* is estimated at 17.3 Ma (95% highest posterior density interval: 12.6–22.8 Ma), and of Australian *Hypseleotris* at 10.1 Ma (7.4–13.4 Ma). These estimates largely agree with those of [11], who also inferred divergence times across Eleotridae based on legacy dates from an earlier fossil-based calibration study [29]. Within *Hypseleotris*, the northwestern clade plus *H. compressa* is dated at 8.9 Ma (6.5–11.7 Ma), with the age of just the northwestern clade at 7.2 Ma (5.3–9.6 Ma). The southeastern radiation (including *H. klunzingeri*) has an estimated crown age of 5.8 Ma (4.4–8.0 Ma), and the hemiclone-forming southeastern species crown age is estimated to be 5.1 Ma (3.8–7.0 Ma). Unfortunately, fossils are as yet unknown for *Hypseleotris* and the related genera examined here, precluding direct fossil calibration of nodes.

**Fig. 5:**
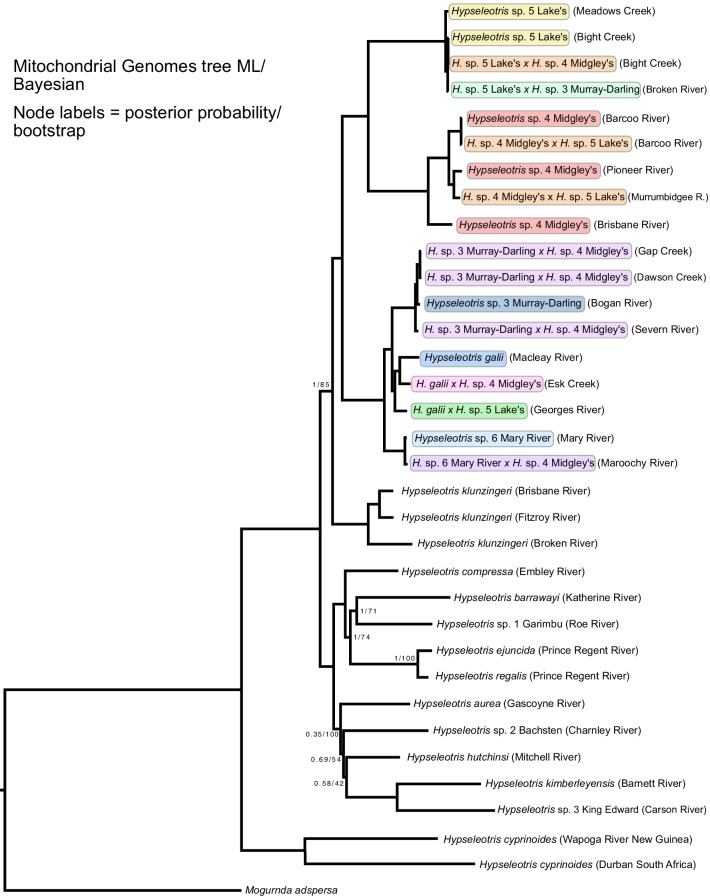
Hypothesis based on analysis of complete mitochondrial genomes. All nodes are supported at 1.0 posterior probability in the Bayesian hypothesis, and 100% bootstrap support in the ML hypothesis, except where indicated; at those nodes the posterior probability/bootstrap support are shown. *Hypseleotris* individuals are labeled in parentheses with the locality where they were collected, not the entire range of the taxon. Colors and names for hemiclone-forming southeastern species are as denoted in Fig. 1

**Fig. 6:**
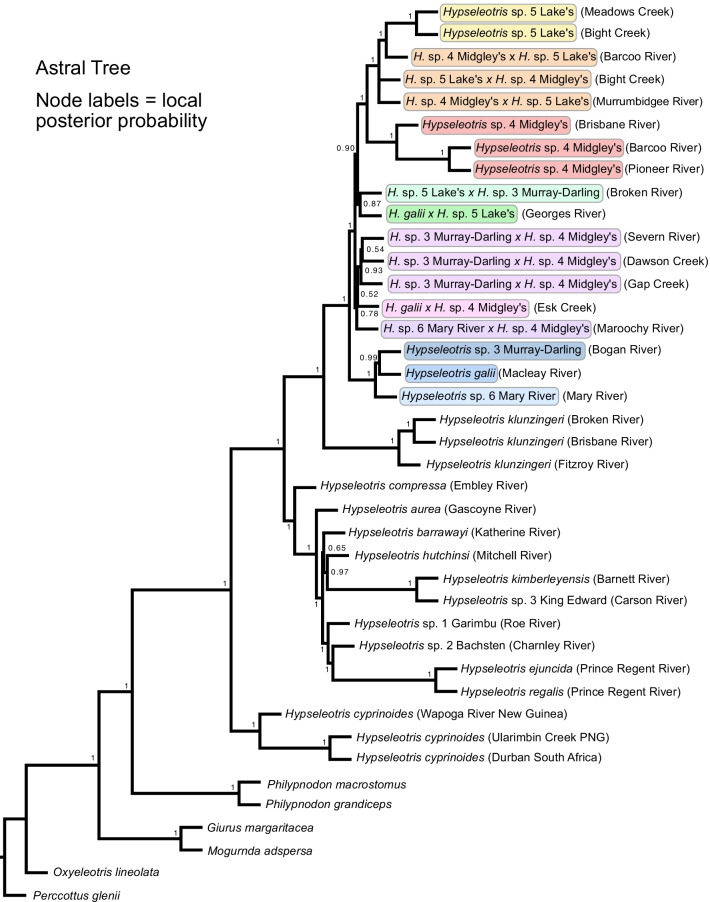
ASTRAL gene tree/species tree analysis. Labels on nodes indicate local posterior probability. *Hypseleotris* individuals are labeled in parentheses with the locality where they were collected, not the entire range of the taxon. Colors and names for hemiclone-forming southeastern species are as denoted in Fig. 1

The phylogenetic hypothesis based on complete mitochondrial genomes is given in Fig. 5. This hypothesis illustrates the mitonuclear discordance found among *Hypseleotris* species, and reveals the maternal ancestry of the hemiclone hybrid pairings. The ASTRAL hypothesis is shown in Fig. 6. The topology differs only slightly from the ML/Bayesian result, placing the *H.* sp. 5 Lake’s × *H.* sp. 3 Murray–Darling and *H. galii* × *H.* sp. 5 Lake’s hemiclones in a slightly different position relative to the other hemiclone pairings, and with lower nodal support. The general stability of the topology other than in the position of the hybrids reveals a lack of incomplete linage sorting among most of the *Hypseleotris* taxa. Hemiclones are expected to have uncertain placement in a bifurcating phylogenetic tree, given their reticulate origin and consistent with our results.

### Regional diversification patterns in Australian *Hypseleotris* clades

**Fig. 7:**
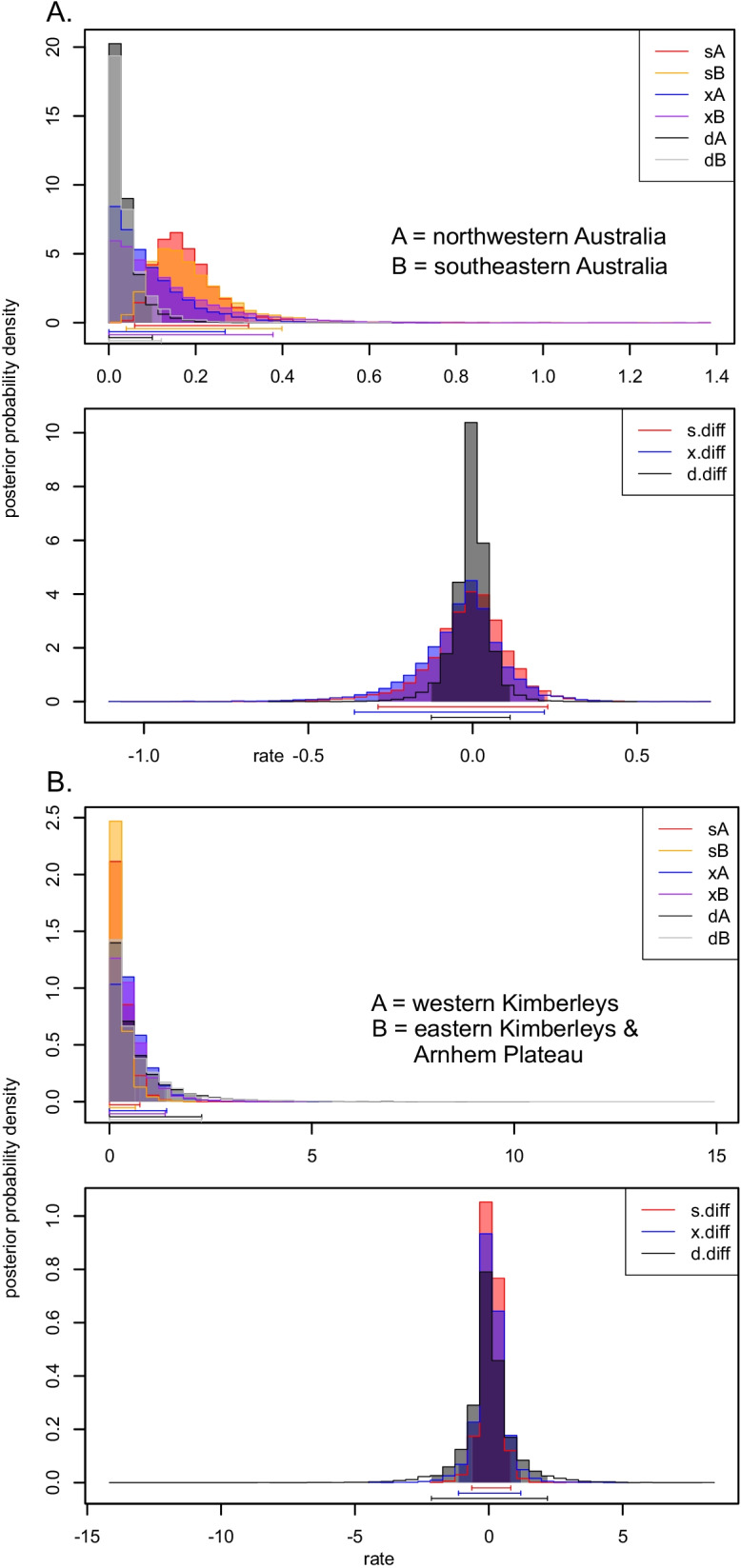
Posterior probability estimates from a Bayesian MCMC analysis of the GeoSSE full model, in which speciation (s), extinction (x), and dispersal (d) are unconstrained between regions. Graphs show values of s, x, and d estimated for each region (upper panel) and the differences in s, x, and d (s.diff, x.diff, d.diff; lower panel) for comparisons of **A** northeastern vs. southeastern Australia and **B** for the western Kimberleys vs. eastern Kimberleys plus the Arnhem Plateau. In both comparisons, s, x, and d do not vary significantly between regions, indicating that diversification rates are steady in *Hypseleotris* clades throughout Australia

Speciation and extinction rates inferred using GeoSSE did not differ significantly between northwestern and southeastern Australian *Hypseleotris* clades. Maximum likelihood estimates of speciation rate derived from the full unconstrained model were 0.117 and 0.104 lineages/Myr for the northwestern and southeastern clades respectively, with extinction estimated at zero in both regions. Estimates of dispersal between the northwest and southeast were also very low, with dispersal out of both regions estimated at zero in the full model. We found no improvement in fit for a model constraining equal speciation and extinction rates versus one in which rates are free to vary (ΔAIC = − 3.96; p = 0.978), and also no improvement in fit for a model disallowing between-region speciation for the northwest and southeast (ΔAIC = − 2.00; p = 1.00). Estimates derived from Bayesian MCMC analysis of the full model without the between-region speciation parameter inferred similar speciation rates: 0.179 and 0.192 lineages/Myr in the northwest and southeast respectively, but higher extinction rates (0.0935 and 0.133 lineages/Myr in the northwest and southeast respectively) and dispersal rates (0.0345 and 0.0390 lineages/Myr in the northwest and southeast respectively). Graphs showing the parameter distributions for the MCMC analysis are shown in Fig. 7.

Finer-scale GeoSSE analysis of just the northwestern species plus *H. compressa* inferred similar speciation and extinction rates between species in the western Kimberleys and eastern Kimberleys plus the Arnhem Plateau. Maximum likelihood estimates from the full model were 0.089 lineages/Myr for speciation in the western Kimberley clade, similar to the speciation rate of 0.082 lineages/Myr in the eastern Kimberley/Arnhem Plateau clade, and extinction rate was estimated at zero in both regions. Estimated dispersal rates between regions were higher at this scale than for the broader Australian comparisons, with dispersal from the western Kimberleys to the eastern Kimberleys/Arnhem Plateau estimated at 0.043 lineages/Myr, and a rate for the reverse (east to west) estimated at 0.030 lineages/Myr. As with the broader Australian analysis, we did not identify a significant improvement in fit for models constraining speciation and extinction between regions (ΔAIC = − 3.99; p = 0.995) or disallowing between-region allopatric divergence (ΔAIC = − 0.795; p = 0.272), although support for between-region allopatry was higher than for the broader northwestern vs southeastern Australia comparison. Bayesian MCMC analysis of the full model without the between-region speciation parameter yielded higher mean estimates of both speciation and extinction; speciation rate in the western Kimberleys was 0.293 lineages/Myr, similar to 0.229 lineages/Myr in the eastern Kimberleys and Arnhem Plateau. Corresponding mean estimates of extinction rates were 0.571 in the western Kimberleys and 0.513 in the eastern region. The mean extinction estimates are higher than the mean speciation rates, implying negative net diversification in both the western Kimberleys and the eastern Kimberleys/Arnhem Plateau. This scenario is possible, but should be viewed with caution given the uncertainty in estimating extinction rates, particularly for small clades such as these. Graphs of parameter estimates from the MCMC analysis are shown in Fig. 7; note that the estimates for both speciation and extinction rates across the Kimberleys are both low, but the extinction estimates have a broader (more uncertain) distribution.

### SNP variant analysis of southeastern *Hypseleotris* species and hemiclones

**Fig. 8:**
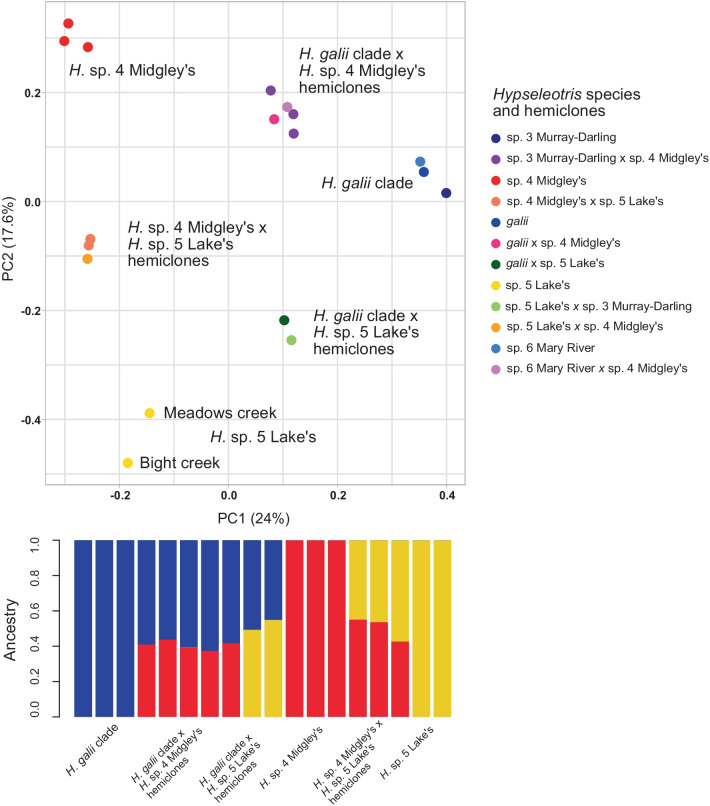
PCA of SNP data (top) and admixture analysis barplot (bottom) for the hemiclone-forming southeastern *Hypseleotris* species. The *H. galii* clade includes *H. galii*, *H*. sp. 3 Murray–Darling, and *H*. sp. 6 Mary River

We recovered 4694 biallelic SNPs from the UCE data for the southeastern species (excluding *H. klunzingeri*) and their hemiclones; PCA of these SNPs is shown in Fig. 8. Patterns recovered here are concordant with an earlier SNP study [23], and show three groups corresponding to the allozyme genotypes: *H.* sp. 5 Lake’s (HX), *H.* sp. 4 Midgley’s (HB) and the *H. galii* clade (HA), along with three intermediate clusters of the hemiclone hybrids. STRUCTURE admixture analysis of the phased SNP data recovers the highest support for K = 3, with demes corresponding to the *H. galii* clade, *H.* sp. 4 Midgley’s, and *H.* sp. 5 Lake’s. Hemiclones exhibit roughly 50% ancestry from each parent, consistent with the hemiclonal hybrid system in which the genetic contributions of the parents do not recombine. Heterozygosity was far higher among the hemiclones (37.1–50.0%) than among the parental species (4.5–14.0%), also consistent with the reticulate ancestry of the hybrids, but note that those values are percentages of heterozygotes among the surveyed SNPs, not the total heterozygosity across the entire nuclear genome.

## Discussion

### *Hypseleotris* phylogeny and the southeastern hemiclones

Our phylogeny (Fig. 3) provides a more comprehensive, robustly-supported hypothesis than has been estimated previously for *Hypseleotris* [21]. The most notable results of our analysis are the recovery of separate clades of *Hypseleotris* species in the northwestern and southeastern regions of Australia, the placement of *H. compressa* as the earliest branching lineage of the clade including the northwestern radiation, and the inclusion of *H. klunzingeri* in the southeastern radiation. We also improve on [21] by obtaining sequence data for all of the species, which in the earlier study was not possible for many of the rarer taxa, and for the species from the remote Kimberley region that were previously unknown. With this more complete sampling we additionally show that *Kimberleyeleotris* is nested within *Hypseleotris* and thus the two *Kimberleyeleotris* species should be transferred to *Hypseleotris,* as denoted in Figs. 3, 4, 5 and 6. The two nominal species of *Kimberleyeleotris* show extremes in some morphological characters (primarily reduced scales and head papillae, atypical presence of a few vomerine teeth, slightly larger mouth) that have been taken to overemphasize their divergence from geographically proximate taxa. This data can equally slightly extend the description or range of counts/measurements for the genus *Hypseleotris* without the need for major review. Unifying characters that support inclusion *Kimberleyeleotris* in synonymy of *Hypseleotris* include pelvic and first dorsal fins placed posteriorly, alignment of second dorsal and anal fins, relatively slender body, urohyal with narrow ventral shelf, metapterygoid slender and separated by long cartilage from quadrate, and eyes laterally placed [16].

The Bayesian and ML phylogenies for the UCE dataset had identical topologies, with the hemiclones in the southeastern clade assorting phylogenetically between their parent taxa (Fig. 3). Analysis of the UCE dataset without the hemiclones resolves the same topology among the included species (Fig. 4). The ASTRAL topology is similar, with some rearrangement among the hemiclones in an overall more poorly-supported part of the tree, but no indication of incomplete lineage sorting among the nuclear UCE loci (Fig. 6). The most markedly different topology is the one based on mitochondrial genomes and shown in Fig. 5, illustrating the mitonuclear discordance that would be expected among the hemiclones, causing them to group with their maternal parents rather than as intermediates between the maternal and paternal lineages.

The hemiclones arising from *H.* sp. 4 Midgley’s and *H.* sp. 5 Lake’s parental taxa may have either species as their maternal parent as both sexes are present among the hemiclones. In this system, unlike standard F_2_ hybrids, once the hemiclone lineage is established the hemiclones appear to only backcross with one of their sexual parent species but then discard the sexual species genotype at meiosis and only produce gametes with the hemiclonal genome [23, 30]. The hemiclones produced between species of the *H. galii* clade (including *H*. sp. 3 Murray–Darling and *H.* sp. 6 Mary River) and *H.* sp. 4 Midgley’s are strongly male biased (96% male for *H.* sp. 3 Murray–Darling × *H.* sp. 4 Midgley’s hemiclones recorded by [14]); they pass the *H.* sp. 4 Midgley’s paternal genome hemiclonally to their offspring and group with their *H. galii* clade parent in the mitochondrial (maternal) phylogeny. Hemiclones between the *H. galii* clade and *H.* sp. 5 Lake’s are strongly female biased (98% female recorded by [14]); their maternal genotype can be that of either sexual parent, and we recover hemiclones of this combination with either the *H. galii* clade or *H.* sp. 5 Lake’s in the mitochondrial phylogeny.

It is unknown if mating between hemiclones does occur; in that event the offspring would either exhibit the genotype of the hemiclonal or the sexual parent, depending on the combination, and the offspring with the sexual parent genotype would not be genetically distinguishable from the parent species. However, there is indirect evidence of hemiclone backcrossing based on comparison of the two individuals identified by multiple genetic datasets as *Hypseleotris* sp. 5 Lake’s. One of the individuals was collected from the restricted range of the pure *H.* sp. 5 Lake’s genotype (Meadows Creek), and that individual is placed in the lower left quadrant of the SNP PCA shown in Fig. 8. The second individual was similarly genotyped as *H.* sp. 5 Lake’s but was collected outside the range of this remnant pure population in Bight Creek, a Kiewa River tributary approximately 360 km to the southwest. The Bight Creek *H.* sp. 5 Lake’s is placed lower in the PCA space, in the region predicted by [23] to be the range of the *H.* sp. 5 Lake’s genotype based on reconstruction from the genotypes of *H.* sp. 5 Lake’s hemiclones. That individual is likely a result of mating between hemiclones, a pairing which is much more likely than that of a hemiclone with the *H.* sp. 5 Lake’s pure parent, given that the range of the pure parent is so restricted while *H.* sp. 5 Lake’s hemiclones are common and widespread. Given the number of individuals sequenced across the Murray–Darling Basin [14, 23] this reestablishment of *H.* sp. 5 Lake’s from two hemiclones is clearly quite rare. It is important to note that [14] also hypothesised one of their hemiclonal combinations was the result of two hemiclones mating.

### Northwestern and southeastern *Hypseleotris* radiations

*Hypseleotris* is remarkable in that it contains two parallel radiations in different parts of Australia with distinct morphologies and ecologies. Clades distributed in the northwest and southeast have a mean crown age of 6.5 Ma (mean 95% HPD 4.9–8.8 Ma) and have diversified in tandem, but in markedly different environments. The northwestern radiation includes nine species (and likely more remaining to be discovered) as opposed to six species (plus likely at least two undescribed lineages within *H. klunzingeri*) with six hybrid combinations in the southeast. While there is inter-specific variation, the northwestern endemic species, especially in the Kimberley region, are proportionally more elongate and slender than their southeastern congeners, with an overall cylindrical body shape (especially as compared to *H.* sp. 4 Midgley’s and *H.* sp. 5 Lake’s and related hemiclones). The southeastern species are almost always sympatric with two to three other species plus their hemiclones, have larger ranges, and usually occur throughout entire drainage systems (with the exception of *H.* sp. 5 Lake’s; Fig. 1). The northwestern taxa typically have small distributions (one to three river drainages) and sympatry has not been observed even with the widespread *H. compressa*, which is only found near the coast [12]. Unlike the southeastern clade, there is no indication that the northwestern species form hybrid lineages.

The earliest branching lineage sister to the northwestern radiation is *Hypseleotris compressa*, a widespread euryhaline species that is found in the Pilbara region of Western Australia, as well as the Kimberley region and coastal rivers around the north and east coast of Australia as far as the New South Wales–Victoria border (Fig. 1). The northwestern radiation is composed of *H. aurea* from the Gascoyne and Murchison rivers (Pilbara region; Fig. 1), followed by eight species (three undescribed) known from restricted ranges in rivers on the Kimberley Plateau (northernmost Western Australia) and Arnhem Plateau (northernmost Northern Territory; Fig. 1). Most are from the Kimberley including: *H. kimberleyensis* (Barnett River, Fitzroy River catchment); *H. ejuncida* (headwaters of the Prince Regent River); *H. regalis* (Roe River and lower half of the Prince Regent River catchment); plus three undescribed species from Garimbu Creek in the Roe River catchment (*H.* sp. 1, Garimbu gudgeon), the Calder River and Charnley River catchments (*H*. sp. 2, Bachsten gudgeon), and Carson River in the King Edward River catchment (*H*. sp. 3, King Edward gudgeon); and a species previously classified in *Kimberleyeleotris*, which we refer to as *H. hutchinsi* (Mitchell River; the congener *H. notata*, known from the Drysdale River, is presumably also included in this clade) [12]. Nested within this group of Kimberley species is *H. barrawayi*, from the nearby Edith and upper Katherine rivers, tributaries of the Daly River catchment in the Northern Territory (Arnhem Plateau). This clade, in contrast to the southeastern *Hypseleotris* clade, is characterized by several short internodes in the phylogeny, consistent with a rapid radiation followed by a long period of isolation.

### Timing of diversification and biogeography

Our inferred divergence dates for *Hypseleotris* clades are given in Fig. 4. Separation of Australian *Hypseleotris* from their widespread, salt-tolerant Indo-West Pacific sister taxon *H. cyprinoides* occurred approximately 17.3 Ma (95% highest posterior density interval: 12.6–22.8 Ma), with the split between southeastern taxa and the clade containing *H. compressa*, *H. aurea*, and the northwestern taxa following at about 10.1 Ma (7.4–13.4 Ma). The presence of two geographically disparate clades in the northwest (with a date for their split at 6.2 Ma [4.5–8.2 Ma]) suggests either two independent invasions into freshwater in that area, or alternatively that extinctions have occurred in the intermediate areas following a single widespread invasion. These dates span the late Miocene, a time when Australia was becoming much drier and cooler, with lowered sea levels relative to the tropical greenhouse conditions present in the early Miocene [22, 31, 32]. In particular, the Pilbara region and the Kimberley and Arnhem plateaus would have been mesic refuges from the overall aridity as their increasing elevation acted to generate orographical rainfall, their topographic complexity reduced evaporation by providing shade, and the non-porose sandstone terrain in the many gorges forced hyporheic flow to the surface [3, 33, 34]. In contrast, the southeast has not experienced more strongly arid conditions due to different rainfall patterns and it is a region of more gentle topography with extensive lowland habitats and far greater opportunities for mixing between river basins.

Speciation within the southeastern and northwestern taxa proceeded throughout the Pliocene and Pleistocene with particular intensity about 5–6 Ma. The northwestern clade features multiple species divergences, mostly in separate river catchments, all occurring in that time frame. The southeastern species diversified at roughly the same time. This period is characterized by an overall cooling trend, punctuated by cyclic glaciations [35]. In Australia, the primary manifestation of these cycles would have been sea level changes, which facilitated vicariant speciation by repeatedly isolating and reconnecting freshwater habitats as they were drowned and exposed by seawater on the continental shelf [36–39]. However, the influence of these fluctuations on rates of vicariant speciation would not have been uniform around Australia’s coastline. Isolation during high (current) sea-levels would be greatest where opportunities for cross-catchment dispersal such as low drainage divides and lowland floodplains are few [2]. Conversely, opportunities for dispersal during lowered sea levels would be greatest where the continental shelf was the widest and rivers had greater opportunities to spread and coalesce [37, 39, 40]

The continental shelf around both the south coast of southeast Australia and northwest Australia is particularly broad and likely facilitated widespread dispersal among freshwater faunas in each region [37, 39]. However the nature of the freshwater environments and degree of freshwater connectivity in each region under current high sea-levels are very different. For instance, many of the rivers in the southeast, particularly those in the Murray–Darling and Lake Eyre basins, take gentle courses through lowland areas, exhibiting muddy channels, ponds, and billabongs that converge into floodplains during wet seasons [3, 41]. Therefore, their fish communities often exhibit broad distributions and patterns of connectivity (e.g. [5, 42–45]). Also, even though the southeastern clade spans four freshwater fish biogeographic provinces (Murray–Darling Basin, Lake Eyre, East Coast, and a very small portion of Northern [2]), several instances of between-province dispersal have been identified, likely resulting from movement across low catchment divides [5, 42–44]. *Hypseleotris* species in this region are widespread, have broadly sympatric ranges and are even partially interfertile to the extent that they have generated hybrid lineages between most of the possible species pairs. Speciation among these taxa could have been allopatric or peripatric early in their evolution, with range expansions occurring easily in the flatter, broadly connected riverine landscapes. Hybrid lineages could then have originally arisen in areas of secondary contact.

Conversely, rivers that drain the Kimberley and Arnhem plateaus in the northwest are highly isolated from one another under current sea-level heights, running more steeply through well-defined rivers characterized by deep sandstone gorges, numerous waterfalls and a lack of flat, lowland areas that would allow for overland connectivity between catchments during floods. Consequently, dispersal between catchments is negligible in all but the most vagile species under current sea-level conditions [39, 46]. These contrasting regional features imply that vicariant allopatric speciation during periods of high sea-level isolation would be the stronger mechanism in the northwest.

Despite their disparate environments and likely speciation mechanisms, we infer no significant shifts in speciation or extinction rates between the northwestern and southeastern radiations across Australia and similarly no support for diversification differences when comparing the western Kimberley and the eastern Kimberley/Arnhem Plateau regions. Speciation and extinction rates are moderate but steady in all regions, although higher extinction and dispersal rates are inferred for the northwestern region in the comparison of taxa inhabiting the Kimberley and Arnhem plateaus. There is no support for between-region speciation (allopatric divergence of an ancestor present in both areas) when comparing larger radiations in the northwest and southeast, which accords with the great distance and topographical differences between the habitats. There is some support (although not statistically significant) for between-region speciation in comparisons of taxa across the Kimberley and Arnhem plateaus. As mentioned above, distributions of species in the Kimberley region and the Arnhem Plateau could be the result of separate invasions of freshwater or from vicariant splits of widespread ancestors (or a combination). The diversification analysis results, in which between-region speciation is not favored, as well as the phylogenetic pattern in which geographically separate clades are geographically isolated (rather than sister taxa occurring in different areas), support the multiple invasion scenario. Within the northwestern radiation are two disjunct *Hypseleotris* clades in the Kimberleys, plus the isolated species *H. barrawayi* on the Arnhem Plateau and *H. aurea* in the Gascoyne and Murchison rivers in Western Australia. Overall, the phylogenetic patterns and diversification estimates suggest that *Hypseleotris* species have repeatedly invaded Australian freshwaters, with incursions occurring as many as six separate times: the broad hemiclone-forming radiation in the southeast, more restricted radiations in the western Kimberleys and eastern Kimberleys, and separately for *H. compressa* in marginal coastal drainages, *H. barrawayi,* and *H. aurea*. *Hypseleotris* species are highly successful colonizers and are now present in all Australian river systems with the exception of the small coastal rivers across the south including Tasmania.

The different characteristics of habitats in the northwest and southeast (steep, isolated drainages in contrast to large, slower flowing rivers) may additionally have contributed to divergence in body forms of some northwestern and southeastern species. Lineages from the Kimberley Plateau are more slender and cylindrical than the robust southeastern species. In the Kimberley rivers the smooth sandstone channels provide little cover, particularly in the wet season when stream flow is much higher. At those times, the fishes can only take shelter in cracks and crevices in the riverbeds or around boulders and stones, favoring a slender morphology. In the southeast, flows are more gentle and aquatic plants and littoral vegetation and debris are more abundant, allowing a more robust body type to succeed [47].

## Conclusions

Our calibrated phylogeny for *Hypseleotris* is the most complete and robustly-supported hypothesis to date. We infer the presence of three clades within *Hypseleotris*: *H. cyprinoides*, the northwestern species plus *H. compressa,* and the southeastern *Hypseleotris* clade. We also show that *Kimberleyeleotris* is nested within *Hypseleotris*. Phylogenies of UCE data inferred using both maximum likelihood and Bayesian methods retrieve a consistent topology, but that topology is discordant with one based on whole mitochondrial genomes extracted from off-target UCE reads. The mitochondrial phylogeny reveals the parentage patterns among the southeastern hemiclones, and SNP data phased from the UCE sequences confirms parentage patterns and reveals that the hemiclones are highly heterozygous.

Using our calibrated phylogeny, we estimate that the primary divergence between Australian *Hypseleotris* clades occurred at roughly 10.1 Ma, followed by a split between two clades in the Kimberley region at approximately 6.2 Ma and the bulk of the speciation in both clades occurring between 5–6 Ma. At this time, Australia was experiencing drier and cooler conditions with lowered sea levels followed by Pleistocene glaciation cycles that would have resulted in sea level variation. These fluctuations could have acted to promote allopatric speciation among river drainages, particularly in the northwestern region where riverine habitats are more isolated. The southwestern species inhabit flatter, more connected landscapes, where peripatric speciation followed by secondary contact (and generation of hybrid hemiclonal lineages) would likely have been the more prominent speciation scenario. Although different speciation mechanisms have likely occurred among *Hypseleotris* species the tempo of speciation and extinction has largely been steady across the continent, with phylogenetic patterns and range-dependent diversification models suggesting multiple freshwater invasions of *Hypseleotris* throughout nearly all of Australian rivers.

There is some future potential to test the generality of our hypotheses regarding the biogeographic and ecological differences between southeast and northwest clades within *Hypseleotris*. A number of other groups among the Australian freshwater fishes, most notably another gudgeon (the genus *Mogurnda*), the rainbowfishes (several genera), the glassfish *Ambassis*, and the atherinid *Craterocephalus*, are speciose (although many candidate species remain undescribed) as well as broadly distributed across southeast, east, north, and northwest Australia [1, 12]. We are currently working towards generating the genomic data required to establish robust systematic frameworks so that such assessments can be made in the future.

## Methods

### Sample collection and UCE sequencing

We assembled a dataset of 40 individuals that encompassed the nine described *Hypseleotris* species, four undescribed southeastern species, three undescribed species from the Kimberley region, representatives of all known hemiclone hybrid crosses, *Kimberleyeleotris hutchinsi*, and six outgroup taxa from the families Eleotridae, Butidae, and Odontobutidae. We collected *Hypseleotris* throughout Australia between 2014 and 2019 using seine nets and euthanized fishes with an overdose of clove oil. For the southeastern *Hypseleotris* species *H.* sp. 4 Midgley’s, *H.* sp. 5 Lake’s, *H.* sp. 3 Murray–Darling*, H.* sp. 6 Mary River, *H. galii,* and their hemiclone hybrids, we confirmed the identity of each individual by both allozyme profiling and by SNP genotypes as described in [23]. For *H.* sp. 5 Lake’s, the pure parental genotype is rare in the wild and is known only from Meadows Creek and nearby Urumwalla Creek, part of the Lachlan River system in New South Wales [23]. We included one individual from Meadows Creek in this analysis, as well as an unusual individual from Bight Creek, a locality near Albury, Victoria that is well to the southwest of the pure parental locality. This individual morphologically resembled pure *H*. sp. 5 Lake’s and exhibited an apparently pure *H.* sp. 5 Lake’s allozyme and SNP genotype.

Specimens of *H. cyprinoides*, the only *Hypseleotri*s species that is not present in Australia but is otherwise widespread throughout the Indo-Pacific, were also collected with seine nets and sampled from New Guinea (Indonesia), Papua New Guinea, and South Africa. We sampled the outgroup taxa *Philypnodon grandiceps* and *P. macrostoma* (Eleotridae) in the same southeastern surveys as the *Hypseleotris*. Samples of *Giurus margaritacea* (Eleotridae), *Mogurnda adspersa* (Eleotridae), *Oxyeleotris lineolata* (Butidae), and *Perccottus glenii* (Odontobutidae) were obtained from the Ichthyology tissue collection at the Natural History Museum of Los Angeles County. Taxa utilized in this study, along with their localities and tissue voucher information, are listed in Table 2.

**Table 2:** Species and hybrids of *Hypseleotris* and outgroups sequenced for ultraconserved elements

Species	Locality	Tissue Voucher
Eleotridae		
* H.* sp. 3 Murray–Darling (M–D)	Bogan River, New South Wales	PU14-122MDCG-1
* H.* sp. 3 M–D × *H.* sp. 4 Midgley’s	Dawson Creek, Angus River, South Australia	SAMA ML-105-1
* H.* sp. 3 M–D × *H.* sp. 4 Midgley’s	Gap Creek, Kiewa River, Victoria	PU13-49MD-5
* H.* sp. 3 M–D × *H.* sp. 4 Midgley’s	Pindari Dam, Severn River, New South Wales	PU14-60MDCG-2
* H.* sp. 3 M–D × *H.* sp. 5 Lake’s	Swanpool Creek, Broken River, Victoria	PU15-94MDCG-F1
* H. aurea*	Gascoyne River, Western Australia	ABTC 68432
* H. barrawayi*	Katherine River, Daly River, Northern Territory	MAGNT A05849
* H.* sp. 4 Midgley’s	Blacks Creek, Pioneer River, Queensland	PU02-44MCG-X
* H.* sp. 4 Midgley’s	Back Creek, Brisbane River, Queensland	PU99-51MCG-1
* H.* sp. 4 Midgley’s	Cooper Creek, South Australia	A12-X
* H.* sp. 4 Midgley’s × *H. galii*	Esk Creek, Brisbane River, Queensland	PU09-90HG-1
* H.* sp. 4 Midgley’s × *H.* sp. 5 Lake’s	Barcoo River, Queensland	PU97-103LCG-1
* H.* sp. 4 Midgley’s × *H.* sp. 5 Lake’s	Wollundry Lagoon, Murrumbidgee River, New South Wales	PU13-42A-LCG-B4
* H.* sp. 4 Midgley’s × *H.* sp. 5 Lake’s	Bight Creek, Kiewa River, Victoria	PU15-94LCG-7
* H.* sp. 4 Midgley’s × *H.* sp. 6 Mary River	Wappa Dam, Maroochy River, Queensland	PU09-100HS-X
* H. compressa*	Trunding Creek, Embley River, Queensland	PU97-88EG-3
* H. cyprinoides*	Wapoga River, New Guinea, Indonesia	LACM T-000140
* H. cyprinoides*	Ularimbin Creek, East Sepik, Papua New Guinea	LACM T-000139
* H. cyprinoides*	Durban, South Africa	LACM T-000141
* H. ejuncida*	Prince Regent River, Western Australia	2D725
* H. galii*	Macleay River, New South Wales	PU99-41HG-1
* H. galii* × *H.* sp. 5 Lake’s	Georges River, Liverpool, New South Wales	SAMA IW94-50-68458
* H.* sp. 5 Lake’s	Meadows Creek, Lachlan River, New South Wales	PU13-3LCG-F9
* H.* sp. 5 Lake’s	Bight Creek, Kiewa River, Victoria	PU15-92LCG
* H. hutchinsi*	Mitchell River, Western Australia	2D218
* H. kimberleyensis*	Barnett River, Fitzroy River, Western Australia	2D078
* H. klunzingeri*	Back Creek, Brisbane River, Queensland	PU99-51WCG-X
* H. klunzingeri*	Vandyke Creek, Fitzroy River, Queensland	PU01-52CG-X
* H. klunzingeri*	Broken River, Victoria	PU13-61HK-1
* H.* sp. 6 Mary River	Kandanga Creek, Mary River, Queensland	PU09-117HG-1
* H. regalis*	Prince Regent River, Western Australia	Z45679
* H*. sp. 1 Garimbu gudgeon	Garimbu Creek, Roe River, Western Australia	Z28943
* H*. sp. 2 Bachsten gudgeon	Charnley River, Western Australia	Z45717
* H*. sp. 3 King Edward gudgeon	Carson River, King Edward River, Western Australia	Z45598
* Giurus margaritacea*	Ross River, Queensland	LACM T-000073
* Mogurnda adspersa*	Ross River, Queensland	LACM T-000130
* Philypnodon grandiceps*	Wollondilly River, New South Wales	PU14-167PG-1
* Philypnodon macrostoma*	Orara River, New South Wales	PU14-53PM.1
Butidae		
* Oxyeleotris lineolata*	Adelaide River, Northern Territory	LACM T-000022
Odontobutidae		
* Perccottus glenii*	Dniestr River, Russia	LACM T-000032

The individual of *H.* sp. 5 Lake’s from Bight Creek displayed the genomic profile of that species but appears to have been derived from a backcross between two hemiclonal parents (see “Discussion”). Tissue voucher numbers with LACM codes are deposited in the fish tissue collection of the Natural History Museum of Los Angeles County, those with MAGNT codes are deposited at the Museum and Art Gallery of the Northern Territory, and those with ABTC (Australian Biological Tissue Collection) codes are deposited at the South Australian Museum, Adelaide, Australia. Those with PU, SAMA, or A field codes are pending accession at the ABTC and may be traced by their field codes and species identifications. Those with field codes beginning with 2 or Z are pending accession at the Ichthyology tissue collection of Museums Victoria, Melbourne, Australia, and may also be traced by their field codes and species identifications

We extracted genomic DNA from tissues using the QIAamp Fast DNA tissue kit (Qiagen Inc., Germantown, MD, USA), quantified DNA concentration with a Qubit 2.0 Fluorometer (Invitrogen Co., Carlsbad, CA, USA), and submitted the extracted DNA to Arbor Biosciences (Ann Arbor, MI, USA) for library preparation, enrichment, and UCE sequencing. We performed two rounds of sample preparation and UCE sequencing, the first using the 0.5Kv1 targeted enrichment probe set (2001 baits for 500 UCE loci) described in [26] and including all of the sexual species. A second round of UCE sequencing included the hemiclone hybrid samples and used the 1Kv1 targeted enrichment probe set (2628 baits for 1341 UCE loci) described in [48]. After completion of library preparation, enrichment, and sequencing, we cleaned reads of adapter contamination and low-quality bases using the parallel *illumiprocessor.py* wrapper for *Trimmomatic* [49]. We used *Trinity* to assemble the quality-trimmed reads into contigs [50], computed assembly coverage using the BWA-MEM algorithm of the Burrows–Wheeler Alignment tool [51] and then trimmed and filtered contigs to a minimum 5× coverage [25, 26] in PHYLUCE [52].

The UCE sequencing was performed in two batches using different targeted enrichment probe sets (0.5Kv1 and 1Kv1). We probed the combined set of contigs twice, once using each of the probe sets. We matched the probes to assembled contigs with minimum coverage and identity threshold parameters set to 80% [26], then tabulated an incomplete matrix of match counts and extracted fasta-formatted UCE sequences for each locus and each taxon. We then aligned UCE sequences with *MAFFT* version 7.4, using the FFT-NS-i algorithm (iterative refinement method) and gap opening penalty of 1.53 [53], cleaned locus names from sequence alignments, and trimmed alignments with gblocks [54]. Finally, we screened alignments for minimum taxonomic coverage requirements of 75 and 95%, and concatenated alignments into sequential format.

To assemble mitochondrial genomes from UCE off-target reads, we followed a procedure similar to that outlined by [55], in which we aligned the *Trinity*-assembled contigs for each species to the mitochondrial genome of *Eleotris acanthopoma* (GenBank accession number AP004455) using the Map to Reference assembly tool in Geneious Prime 2020.0.5 (www.geneious.com). We transferred annotations from the *E. acanthopoma* genome across the assembled mitochondrial contigs, and aligned the genomes using the MAFFT multiple alignment tool, using the FFT-NS-i algorithm (iterative refinement method) and gap opening penalty of -2.0, also implemented in Geneious Prime.

### Phylogenetic inference and calibration

For the UCE data, the 95% taxon complete coverage matrices obtained by probing contigs with 0.5Kv1 and 1Kv1 targeted enrichment probe sets resulted in very small matrices, each with 15 loci and roughly 25,800 base pairs. The 75% taxon complete coverage matrices were much larger, with 383,551 base pairs for 238 loci with the 1Kv1 probe set and 412,583 base pairs for 251 loci with the 0.5Kv1 probe set. Most of those loci were overlapping, so we proceeded with the larger 251 locus dataset for all subsequent phylogenetic analyses (we refer to this as the UCE dataset). After alignment and trimming, the 251 loci in the UCE dataset ranged from 802 to 2854 base pairs in length (95% CI ± 31). The matrix of mitochondrial genomes was composed of 16,729 base pairs and included 34 taxa, representing all of the *Hypseleotris* species and hemiclones apart from one *H. cyprinoides* sample which did not yield complete coverage of the mitochondrial genome. Similarly, among the outgroup taxa only *Mogurnda adspersa* yielded a complete genome from the off-target reads, so only that species was used as the outgroup in phylogenetic analyses. We refer to this matrix as the mitochondrial dataset.

To estimate phylogeny, we performed partitioned maximum likelihood (ML) phylogenetic analyses on both the UCE and mitochondrial datasets using RAxML 8.2.12 [56] at the CIPRES science gateway at phylo.org, using the XSEDE supercomputer [57]. We included hemiclone hybrid individuals in analyses of the UCE dataset even though such taxa violate the assumption of no reticulation among lineages, in order to explore their placements relative to their parental species. For the mitochondrial phylogeny, the assumption of a strictly bifurcating pattern is not violated in that the mitochondrion traces only the maternal lineages and does not recombine. For the nuclear UCE data, however, the hemiclones include one hemiclonally transmitted haploid genome, combined with a haploid genome from one of their sexual parent species. The genomes do not recombine, but even so, each individual is the product of reticulate ancestry. Therefore, we performed both ML and Bayesian analyses separately on a reduced UCE dataset that did not contain the hemiclone hybrids, and also without one of the *H.* sp. 5 Lake’s individuals that we suspect is the result of a hemiclone backcross (detailed in the “Discussion”). We used this hypothesis for calibration and estimation of divergence times.

We partitioned the data for all phylogenetic analyses by assigning separate data partitions for each UCE locus or mitochondrial gene, and then evaluated partitioning schemes using PartitionFinder 2.1.1, with the rcluster search algorithm and GTRGAMMA model and evaluated for fit with the corrected Akaike information criterion [58, 59]. This resulted in 127 partitions for the UCE alignment and 14 partitions for the mitochondrial genome alignment. In each of these partitioned analyses, we applied the GTRGAMMA substitution model and assessed support for nodes in each RAxML tree with non-parametric bootstrapping set to finish based on the autoMRE majority rule criterion.

For Bayesian phylogenetic analysis, also on both the UCE (with and without hemiclones) and mitochondrial datasets, we used the multi-threaded, MPI hybrid version of *ExaBayes* to sample the Bayesian posterior distribution of phylogenetic trees with MCMC simulations [60], also implemented at the CIPRES science gateway at phylo.org and using the XSEDE supercomputer [57]. We executed four independent runs of 1 million generations each with random starting trees, drew samples from the Bayesian posterior distribution of trees every 500 generations, and used the default average standard deviation of split frequencies (ASDSF) topological convergence threshold of 5% or less. We used the *consense* and *credibleSet* software distributed with *ExaBayes* to compute 50% majority rule consensus trees and credible tree sets, respectively [60], and examined run parameters with Tracer 1.7 [61], to confirm that effective sample size (ESS) values for all parameter estimates exceeded 200.

To investigate potential gene-tree/species-tree discordance due to incomplete lineage sorting we analyzed the complete UCE dataset, constructing individual gene trees for each UCE locus and summarizing them into a species tree using ASTRAL. To construct the gene trees, we decomposed the concatenated alignment into alignments for each UCE locus using RAxML, removed taxa with only missing data using trimAl [62], and then again used RAxML to estimate phylogeny for each locus with the GTRGAMMA substitution model and nodes with less than 10% branch support collapsed. We then used ASTRAL5.6.3 [63, 64] to summarize the individual gene trees into a species tree under the multispecies coalescent model with branch support measured as local posterior probability.

As discussed above, we used the UCE phylogeny based on a reduced dataset (without hemiclone hybrids) for time-calibration with MCMCTree, as implemented in PAML 4.8 [65, 66] and using the two-step approximate likelihood calibration procedure of [67]. We applied the independent rates clock model, GTR + gamma substitution model, and a root age estimate of 53.9 Ma (derived from age of Butidae + Eleotridae in [11]). We ran the analysis three times, each with 1,000,000 generations and a 10,000 generation burnin, and checked for convergence by comparing the posterior mean times of each run against the other two. We applied three calibrations: root of Eleotridae at 45.5 Ma (95% highest posterior density interval: 34.0–57.6 Ma), divergence of *Giurus margaritacea* and *Mogurnda adspersa* at 19.3 Ma (10.0–29.1 Ma), and root of Australian *Hypseleotris* (excluding *H. cyprinoides*) at 9.0 Ma (4.3–15.8 Ma). These dates are legacy calibrations derived from fossil-based calibrations in [11] and [29], and we applied them as ranges corresponding to the 95% confidence intervals of the estimated ages.

### Analysis of range-dependent diversification patterns

To determine whether or not the *Hypseleotris* radiations in different geographical areas exhibited shifts in diversification rate, we used the calibrated phylogeny to compare diversification dynamics of clades in northwestern and southeastern Australia with GeoSSE [68]. To avoid comparisons to distant outgroups and enable coding of species range as three states (northwestern [including *H. aurea*], southeastern, and widespread Australian [*H. compressa*]) as required by GeoSSE, we used the R package *ape* version 5.0 [69] to reduce the calibrated phylogeny to Australian species of *Hypseleotris* only. We then used treePL [70] to generate an ultrametric tree, using an age estimate for Australian *Hypseleotris* of 7.5–13.3 Ma (the 95% confidence interval inferred in the calibrated MCMCtree phylogeny), and performed range-dependent diversification analyses with GeoSSE as implemented in the R package *diversitree* version 0.9-16 [71]. We inferred maximum likelihood estimates of speciation, extinction, and dispersal rates for each region, plus a parameter estimating between-region speciation in which a single widespread species in both regions splits into separate species in each region. We then fit models estimating these parameters for three scenarios: unconstrained, with all rates permitted to vary by region; constrained for equal speciation and extinction between regions; and constrained to prohibit between-region speciation. We compared model fits for the unconstrained and constrained models with Akaike information criteria (AIC) and evaluated significance using a χ^2^ test. Significantly worse fit for the constrained equal diversification model would indicate that diversification rates differ between regions. Significantly worse fit for the model prohibiting between-region speciation would indicate that between-region speciation (splits of widespread ancestral lineages) is a significant mode of diversification, in contrast to speciation mostly occurring within the regions. In order to visualize the probability density of the estimates for speciation, extinction, and dispersal, we additionally performed a Bayesian MCMC analysis of the model with the between-region speciation parameter omitted.

We performed a second GeoSSE analysis focusing just on the northwestern species plus *Hypseleotris compressa* to compare the diversification rates of sister clades in the Kimberley region and to evaluate the hypothesis that their distribution is the result of divergence of a widespread ancestor (between-region speciation following a single invasion of freshwater) rather than within-region speciation likely tied to multiple independent freshwater invasions. One clade, including *H. ejuncida*, *H. regalis*, *H*. sp. 1 Garimbu and *H*. sp. 2 Bachsten, is distributed in a small portion of the western Kimberleys encompassing the Roe, Prince Regent, Calder, and Charnley rivers, all with relatively short courses that drain to the ocean in the same general area. The second clade includes *H. barrawayi*, *H. hutchinsi*, *H. kimberleyensis*, and *H*. sp. 3 King Edward. That clade is distributed across a wider area to the east of its sister clade, particularly *H. barrawayi* which inhabits the Katherine River on the Arnhem Plateau in the Northern Territory, far to the east of the others. As with the broader Australian GeoSSE analysis, we first reduced the calibrated phylogeny to only the northwestern species and the widespread *H. compressa*. In addition to the Kimberley clade, the northwestern species include *H. aurea*, a species known from the Gascoyne and Murchison rivers in central Western Australia that is disjunct and distant from the species in the Kimberley region. Because GeoSSE does not permit coding of more than two areas (plus the “widespread” state which inhabits both), we could not code *H. aurea* as western Kimberley or eastern Kimberley/Arnhem Plateau and instead coded it as widespread along with *H. compressa*. (Similar results were obtained in an analysis performed with *H. aurea* removed). We then fit constrained and unconstrained models as described above and evaluated model fits using Akaike information criteria (AIC) and χ^2^ tests, as well as a Bayesian MCMC analysis of the model without the between-region speciation parameter. It has been shown that state-dependent diversification models (including GeoSSE) may be biased in large phylogenies to yield high rates of type I error, falsely supporting differences in diversification rate and requiring correction of significance thresholds to adjust for such bias [72]. Our analyses involved small clades with complete sampling, and as we did not recover evidence for any diversification shifts, corrections to significance thresholds were not required.

### Extraction and phasing of SNPs from UCE data

To further investigate genetic patterns among the southeastern hemiclones, we extracted and phased SNPs out of the UCE data for the southeastern species *Hypseleotris galii*, *H.* sp. 3 Murray–Darling, *H.* sp. 4 Midgley’s, *H.* sp. 5 Lake’s, *H.* sp. 6 Mary River, and all the known hemiclone combinations arising from those parent species, a total of 19 individuals among our samples. For SNP phasing, we mapped the quality-trimmed reads from the phylogenetics pipeline against the reference genome of a related species, the mudskipper *Boleophthalmus pectinirostris* (NCBI Assembly GCF_000788275.1) [73] using BWA-MEM [51]. We sorted, removed duplicates, and added read groups for the assemblies with Picard (http://broadinstitute.github.io/picard/) and converted SAM files to BAM with SAMtools [74]. Then, we used the Genome Analysis Toolkit (GATK) [75] to call SNPs for each individual and combine the individual variant call files (VCF) into a single multisample file. We filtered the multisample VCF file for biallelic SNPs with < 10% missing data, a minimum quality score (minQ) of 30 and a minor allele frequency (maf) of > 10% using VCFtools [76].

To explore the pattern of variation in the SNPs, we used PLINK [77] to extract eigenvectors from the VCF file and plotted the results using the *tidyverse* package in R 4.0.5 [78, 79]. We also performed an admixture analysis using fastSTRUCTURE 1.0 [80] and generated a bar plot in R. Finally, we extracted the number of heterozygotic SNPs for each individual from the VCF file using BCFtools stats [74].

## Data Availability

The datasets generated and/or analysed during the current study are available in the NCBI SRA repository under BioProjects PRJNA774634 (*Hypseleotris* species), PRJNA348720 (*Mogurnda adspersa*) and PRJNA659476 (remaining outgroups).
